# Polycomb Repressive Complex(es) and Their Role in Adult Stem Cells

**DOI:** 10.3390/genes12101485

**Published:** 2021-09-24

**Authors:** Pooja Flora, Gil Dalal, Idan Cohen, Elena Ezhkova

**Affiliations:** 1 Department of Cell, Developmental, and Regenerative Biology, Black Family Stem Cell Institute, Icahn School of Medicine at Mount Sinai, 1 Gustave L. Levy Place, New York, NY 10029, USA; pooja.flora@mssm.edu; 2 The Shraga Segal Department of Microbiology, Immunology and Genetics, Faculty of Health Sciences, Ben-Gurion University of the Negev, Beer Sheva 84105, Israel; gilda@post.bgu.ac.il

**Keywords:** adult stem cells, histone modifications, polycomb complexes, PRC1, PRC2

## Abstract

Populations of resident stem cells (SCs) are responsible for maintaining, repairing, and regenerating adult tissues. In addition to having the capacity to generate all the differentiated cell types of the tissue, adult SCs undergo long periods of quiescence within the niche to maintain themselves. The process of SC renewal and differentiation is tightly regulated for proper tissue regeneration throughout an organisms’ lifetime. Epigenetic regulators, such as the polycomb group (PcG) of proteins have been implicated in modulating gene expression in adult SCs to maintain homeostatic and regenerative balances in adult tissues. In this review, we summarize the recent findings that elucidate the composition and function of the polycomb repressive complex machinery and highlight their role in diverse adult stem cell compartments.

## 1. Introduction

Adult stem cells (SCs) are a special population of undifferentiated cells that reside within specific tissues or anatomic locations to maintain tissue homeostasis throughout life, by constantly replenishing damaged cells [1]. How adult SCs maintain cellular identity and balance between their ability to self-renew and generate specialized progenitor cells within a tissue is a subject constantly under investigation. Among the various mechanisms controlling SCs, epigenetic regulation of transcriptional programs has emerged as a key mechanism underlying the formation and maintenance of cellular identity and tissue-specific expression patterns [2]. In addition, epigenetic regulation of adult SCs allows for a differential utilization of the same genetic information to balance between self-renewal or differentiation. 

Among epigenetic factors, the PcGs of proteins represent an evolutionarily conserved mechanism that plays a central role in regulating cellular identity and developmental programs in higher eukaryotes [3]. Initially discovered through genetic screens for homeotic transformation in *Drosophila melanogaster*, PcG proteins were shown to act as repressors of homeotic genes, thereby ensuring their spatiotemporal gene activation and correct pattern formation in the developing *Drosophila* [4,5,6,7]. PcG regulation of developmental genes is also conserved in the mammalian system. In embryonic stem (ES) cells, PcG proteins play an instrumental role in maintaining an undifferentiated state by silencing key developmental regulators [8,9,10,11,12]. Moreover, PcG components are required for embryonic development past the gastrulation stage [13,14,15,16]. In adult SCs, the PcGs of proteins plays various roles, including the regulation of cell differentiation, cell proliferation, and survival states [17,18,19,20,21,22,23,24,25,26]. In this article, we review the recent advances in the understanding of the polycomb machinery complex composition and functions, including the surprising identification of non-canonical polycomb complexes at active genes, and focus on their diverse roles in adult tissue SCs.

## 2. Classification of Mammalian Polycomb Complexes

Initial biochemical studies in *Drosophila* have shown that different PcG proteins assemble to form two main functionally distinct multimeric complexes, termed based on their well-known transcriptional repressor activity as polycomb repressive complex 1 (PRC1) and 2 (PRC2) [27]. PRC1 contains an E3 ubiquitin ligase activity and catalyzes histone H2A lysine 119 mono-ubiquitination (H2AK119ub) [28,29,30], whereas PRC2 possesses a methyltransferase activity and catalyzes histone H3 lysine 27 mono/di/tri-methylation (H3K27me1/2/3) [31,32,33,34,35]. Usually, PRC1 and PRC2 colocalize at the genome to a great extent, where their shared binding and cooperation enforce transcriptionally silent polycomb domains marked by the H2AK119ub and H3K27me3 histone modifications [12,36,37,38,39]. Historically, it was believed that polycomb complexes function via a simple hierarchical model, where PRC2-mediated H3K27me3 acts as a cue to recruit PRC1, which in turn catalyzes H2AK119ub deposition and chromatin compaction [29,40,41]. However, data from recent studies suggest that the functions and diversity of polycomb complexes are greater than originally anticipated [42,43,44]. Indeed, while the core components of polycomb complexes are highly conserved during evolution, mammalian polycomb complexes are more diverse and can interact with multiple accessory subunits. This diversity potentially renders each complex active in various molecular functions [44,45,46], including the contrasting and puzzling observations of some polycomb complex components at active loci [47,48,49,50].

### 2.1. PRC1 Complexes Form Canonical and Non-Canonical Complex Assemblies

**Figure 1:**
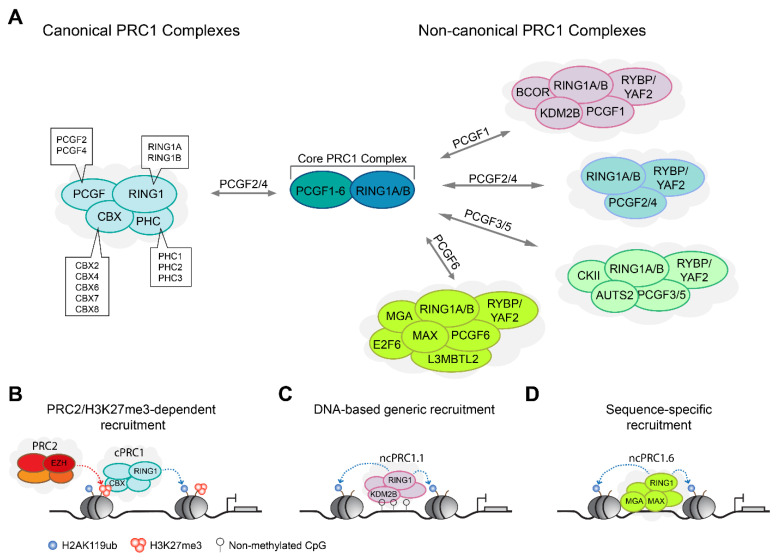
Composition of PRC1-type complexes. (**A**) PRC1 complexes are divided into two main groups, canonical PRC1 (cPRC1) complexes and non-canonical (ncPRC1) complexes. Both groups contain, at their core, a PCGF subunit, together with an E3 ubiquitin ligase subunit RING1A or RING1B that catalyzes H2AK119ub. cPRC1 complexes are restricted to PCGF2 or PCGF4 and are defined by the presence of PHC protein and CBX protein that can recognize PRC2-mediated H3K27me3. ncPRC1 complexes lack CBX and PHC proteins and instead contain RYBP/YAF2 proteins, and their cores can be formed with any of the PCGF1-6 proteins. (**B**–**D**) Illustration of three major mechanisms for PRC1 recruitment. (**B**) A PRC2-dependent mechanism in which cPRC1 complexes are recruited to chromatin via H3K27me3 reader activity of CBX proteins. (**C**) A DNA-based generic recruitment of ncPRC1 complexes via KDM2B binding activity to genomic regions enriched for CpG islands. (**D**) A sequence-specific recruitment of ncPRC1 complexes via interaction with transcription factors.

Mammalian PRC1 complexes are extremely diverse in their core and accessory subunit composition, and therefore potentially possess different biochemical activities in each complex subtype. All PRC1-type complexes contain at their heteromeric core an E3 ubiquitin ligase, RING1A or RING1B, together with one of the six polycomb group RING finger (PCGF) subunits [29,30,44]. The RING finger domain of the RING1A/B is important for protein–protein interaction with PCGF subunits, which together form the minimal PRC1 core required for the catalysis of H2AK119ub [44,51,52,53]. The presence of a specific PCGF protein (PCGF1 to PCGF6) is used to name and distinguish between the different PRC1 complexes, ranging from PRC1.1 to PRC1.6 [44]. In addition, RING1A/B and PCGF proteins each contain a ring finger structure and a WD40-associated ubiquitin-like (RAWUL) domain, which forms essential contacts with other PRC1 subunits that confer a more specific biochemical property to the assembled complexes [54,55,56]. At large, PRC1 complexes can be divided into two major groups, termed canonical PRC1 (cPRC1) and non-canonical PRC1 (ncPRC1) (Figure 1A). Below, we describe the main PRC1 complexes.

All cPRC1 complexes are assembled around PCGF2 (also known as MEL18) or PCGF4 (also known as BMI1) and are defined by the presence of a CBX protein [36,44]. For reasons unknown, other PCGF subunits do not form cPRC1 complexes. In addition to the RING1A/B and PCGF2/4 core, cPRC1 complexes contain one of three PHC subunits (PHC1-3), and one of the five CBX subunits (CBX2/4/6/7/8) [28,57,58,59] (Figure 1A). The CBX proteins are readers of the PRC2-mediated H3K27me3 mark [40,41,60], thus providing a molecular link between PRC1 and PRC2 (Figure 1B). The ability of cPRC1 complexes to recognize H3K27me3, together with the observations that loss or manipulation of H3K27me3 levels affects PRC1 genomic binding, has led to the hypothesis that PRC2-mediated recruitment and H3K27me3 deposition signals to recruit PRC1 [12,34,40,61]. However, this notion changed with the discovery of ncPRC1 complexes that can be recruited to chromatin independently of PRC2 or H3K27me3 [62,63,64]. 

Mammalian ncPRC1 complexes lack CBX proteins and instead contain RYBP, or its homologue YAF2, which assemble with PCGF1–PCGF6 to form ncPRC1.1–ncPRC1.6, respectively [44]. The presence of different PCGF subunits affects the association with additional accessory subunits, as each of these ncPRC1 complexes assembles different accessory subunits. For example, the presence of PCGF1 within ncPRC1.1 complexes enables the association with BCOR and the H3K36 histone lysine demethylase KDM2B (also known as FBXL10) [64,65,66]. KDM2B contains a CXXC-zinc finger domain that recognizes unmethylated CpG islands and thus contributes to generic genomic localization of PRC1 around promoters and other loci enriched for unmethylated CpG islands [64,67,68,69,70,71] (Figure 1C). On the other hand, the presence of PCGF6 within ncPRC1.6 complexes enables the association with E2F6, MAX, and MGA transcription factors that contribute to the context-specific recruitment of PRC1 to genomic loci [72,73,74,75] (Figure 1D). While it is not fully known to what extent the various ncPRC1 complexes localize to distinct vs overlapping genomic regions [44,47,76], the different subunit composition and biochemical properties gained by the assembly of various accessory subunits suggests functional diversity. 

### 2.2. PRC2 Complexes Form Type 2.1 and Type 2.2 Complex Assemblies

**Figure 2:**
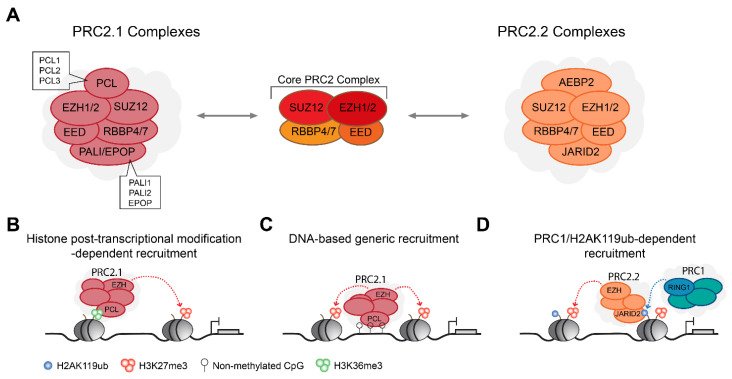
Composition of PRC2-type complexes. (**A**) PRC2 complexes are divided into two main groups, PRC2.1 and PRC2.2. Both groups contain at their core RBBP4 or RBBP7, EED, SUZ12, and a histone methyltransferase subunit EZH1 or EZH2 that catalyzes H3K27me3. The PRC2.1 complex contains a PALI or an EPOP subunit, and a PCL protein that recognize the H3K36me2/3 histone marks. The PRC2.2 complex contains AEBP2 protein and a JARID2 protein that recognize PRC1-mediated H2AK119ub. (**B**–**D**) Illustration of three major mechanisms for PRC2 recruitment. (**B**) Histone modifications can mediate PRC2.1 recruitment via H3K36me3 reader activity of PCL proteins. (**C**) Similar to PRC1, PRC2.1 complexes also display a DNA-based generic recruitment via PCL proteins winged-helix domain binding activity to genomic regions enriched for CpG islands. (**D**) PRC2 recruitment can also be mediated in a PRC1-dependent manner, through H2AK119ub reader activity of the core PRC2.2 subunit JARID2.

Mammalian PRC2 complexes are relatively homogenous at their core when compared with PRC1, and contain, at their heteromeric core, a SET domain containing histone methyltransferase, EZH1 or EZH2, together with EED and SUZ12 [33,34]. Similar to RING1A/B, EZH1 and EZH2 are also mutually exclusive in the assembled PRC2 complexes [77,78]. All three core subunits are required for the methyltransferase activity of EZH1/2 towards the catalysis of H3K27me1/2/3 [31]. The EED subunit can recognize H3K27me3 and thus contributes to genomic self-propagation of the PRC2 complex and H3K27me3 deposition [79,80]. A fourth core subunit of the histone binding proteins, RBBP4 or RBBP7, is important for the PRC2 complex genomic stabilization [81,82,83]. Additional biochemical analyses have revealed that PRC2 complexes can be further divided into two mutually distinct PRC2 complexes termed PRC2.1 and PRC2.2 [77,84], depending on the assembly of additional accessory subunits (Figure 2A). 

The PRC2.1 complex is defined by the assembly of one of the three polycomb-like protein 1-3 (PCL1-3), either one of the two polycomb associated LCOR isoform 1/2 (PALI1/2) or elongin B/C and PRC2-associated protein (EPOP) [46,77]. PCL proteins contribute to PRC2 genomic targeting via their TUDOR domain that can recognize the H3K36me2/3 histone marks associated with transcriptional elongation [85,86,87,88] (Figure 2B), while EPOP and PALI proteins modulate PRC2 histone methyltransferase enzymatic activity [46,89,90,91,92]. In addition, PCL proteins contain a winged-helix domain that can recognize unmethylated CpG islands [93,94,95], thus facilitating generic genomic localization of PRC2 around loci enriched for unmethylated CpG islands (Figure 2C).

The PRC2.2 complex contains two main accessory subunits, the Jumanji AT-rich interacting domain 2 (JARID2) protein and the adipocyte enhancer-binding protein 2 (AEBP2). Mechanistically, JARID2 is an atypical member of the Jumanji family of transcriptional regulators that lacks histone demethylase activity [96,97]. Instead, JARID2 plays two main roles in PRC2 activity; first to stabilize PRC2 genomic binding, a function which may be in part due to its ability to recognize the PRC1-mediated H2AK119ub histone mark [98,99,100] (Figure 2D), and second, the phosphorylation of JARID2 at lysine 116 stimulates PRC2 catalytic activity [101]. AEBP2 was also reported to regulate PRC2 catalytic activity together with JARID2 [102,103], yet its precise role within the PRC2 complex is not fully understood.

## 3. Transcriptional Regulation by Polycomb Complexes

Transcriptional regulation by polycomb complexes plays important roles in cellular and developmental processes and relies not only on their histone modification activity but also on various non-catalytic activities mediated by the different accessory subunits [42,104]. Here we discuss the main molecular mechanisms by which polycomb complexes repress transcription. 

### 3.1. Role of PRC1 Catalysis and H2AK119ub

While all PRC1 complexes contain the RING1A/B ubiquitin ligase capable of catalyzing H2AK119ub, variable proficiency in the catalytic activity has been reported based on the particular PRC1 complex’s composition. Specifically, the RYBP/YAF2 subunit in ncPRC1 complexes seems to play an important role in stimulating the E3 ubiquitin ligase activity of RING1A/B. Molecular in vitro studies comparing the E3 ligase activity of PRC1 minimal core (PCGF-RING1B) demonstrated that the interaction of RYBP with the minimal PRC1 core further stimulates its enzymatic activity [44,63,105]. In contrast, the interaction of a PRC1 minimal core composition with ncPRC1 subunits such as KDM2B or BCOR, or cPRC1 subunits such as CBX7, CBX8, and PHC2, exhibited weaker catalytic activity when compared to RYBP-containing PRC1 minimal core complex [63,105]. Within ncPRC1 complexes, increasing evidence from several experimental systems further implies that a large portion of H2AK119ub deposition is catalyzed by ncPRC1.3 and ncPRC1.5 complexes, as the loss of PCGF3/5 in ES cells or epidermal progenitor cells resulted in a drastic reduction in the global levels of H2AK119ub [47,76,106]. However, additional studies are needed to determine the exact contribution of specific ncPRC1 complexes to H2AK119ub in different cell types and tissues. 

The importance of PRC1 catalytic activity and H2AK119ub in transcriptional regulation was a matter of debate and their central role in transcriptional repression only recently come to light (review in Cohen et al.) [28]. Indeed, studies of PRC1 mutants in the skin epidermis using a RING1A-null background with a conditional RING1B mutant (I53A) demonstrated a central role for PRC1 catalytic activity in transcriptional repression in vivo. [28]. It is important to mention, however, that a stronger effect was observed upon global loss of PRC1 activity, indicating that non-catalytic activity of PRC1 also plays a role in transcriptional repression. Significantly, the loss of PRC1 catalysis in epidermal progenitors during development recapitulated the PRC2-null phenotype, demonstrating de-repression of Merkel cell developmental factors and resulted in an ectopic formation of mechanosensory Merkel cells throughout the skin epidermis [47,107,108]. Finally, the central role of PRC1 catalysis in transcriptional repression was recently demonstrated in ES cells. Since molecular studies suggested that the I53A point mutation in RING1B may be hypermorphic, Blackledge and colleagues used a double-mutant RING1B (I53A/D56K) completely lacking residual E3 ubiquitin ligase activity. This experimental setting demonstrated that loss of PRC1 catalytic activity leads to a depletion of H2AK119ub and similar defects in gene expression when compared to PRC1-null ES cells [109]. Taken together, these studies provide strong evidence of prominent roles played by PRC1 catalysis and H2AK119ub in transcriptional repression. 

**Figure 3:**
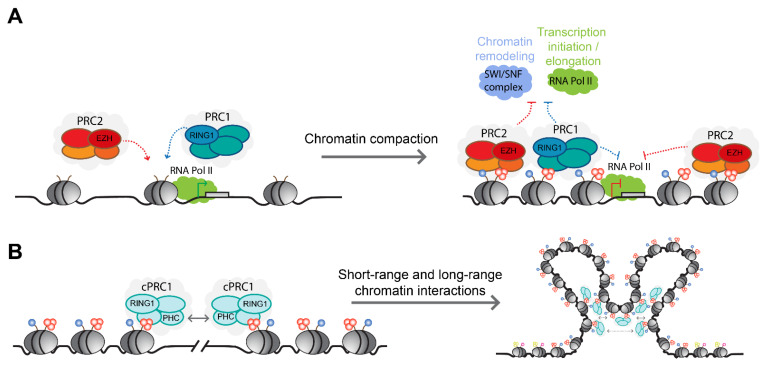
Transcriptional regulation by polycomb complexes. (**A**) Schematic illustration of polycomb-mediated transcriptional repression. Polycomb activity affects transcription at multiple levels, by compacting chromatin and limiting accessibility of chromatin remodeling complexes and transcription factors, as well as by blocking and interfering with RNA Pol II activity. (**B**) Polymerization activity by the SAM domain of the cPRC1 subunit PHC, which promotes chromatin looping and long-rang interactions that contribute to the transcriptional silencing and stable genomic binding of polycomb complexes.

How the PRC1 catalysis of H2AK119ub regulates transcription is not fully understood. PRC1-mediated H2AK119ub was originally proposed to interfere with RNA polymerase II activity by affecting the assembly/stability of the pre-initiation complex, or by blocking the release of paused RNA polymerase II and transcriptional elongation [110,111,112,113] (Figure 3A). In addition to PRC1 roles in blocking transcriptional initiation and elongation, recent studies challenging the main dogma of polycomb-mediated transcriptional control demonstrated that PRC1 catalysis of H2AK119ub plays an important role in the recruitment of PRC2 complexes. A subtype of PRC2, PRC2.2 complexes, can recognize H2AK119ub via their JARID2 and AEBP2 subunits, and loss of PRC1 catalysis and H2AK119ub have been shown to reduce PRC2 core subunits genomic binding and H3K27me3 deposition [47,98,109,114,115]. In turn, such a reduction can also impair the binding of cPRC1 complexes that promote chromatin compaction and transcriptional repression via long-range chromatin interactions between polycomb-marked loci. Alternatively, H2AK119ub could recruit other reader proteins with transcriptional repressor activity, or directly block or interfere with the deposition of histone modification associated with transcriptional activation. It is also possible that PRC1 catalysis plays an indirect role through the post-transcriptional modification of non-histone substrates. 

### 3.2. Role of PRC2-Mediated H3K27me3

The deposition of H3K27me3 by PRC2 complexes varies depending on the identity of the histone methyltransferase core subunit EZH1 or EZH2. The majority of H3K27me3 deposition is attributed to EZH2 activity, which is essential for mammalian embryonic development and is enriched in proliferating cells such as ES cells, myoblasts, epidermal progenitors, and hematopoietic stem/progenitor cells. EZH1, on the other hand, has a reduced histone methyltransferase activity, is dispensable for mammalian embryonic development, and is assumed to act in mature or resting cells [15,50,78,99,116,117]. The differences between these two catalytic core subunits stem from the nature of their interaction with the PRC2 core subunit EED, which mediates the allosteric activation of EZH1/2 [118]. In addition, PRC2 catalytic activity is largely through interactions with accessory subunits. For example, the presence of EPOP in PRC2.1 complexes stimulates a moderate deposition of H3K27me3, while the presence of AEBP2 and JARID2 in PRC2.2 complexes allosterically stimulates PRC2 catalytic activity and leads to enhanced H3K27me3 deposition [45,90,91,99,101,102,119]. JARID2 also binds to PRC1-mediated H2AK119ub, thus providing a functional link between the two main forms of polycomb complexes [98,114]. Conversely, the interaction with EZHIP (also known as CATACOMB) inhibits PRC2 catalytic activity and leads to reduced H3K27me3 levels [120,121,122]. 

The importance of the H3K27me3 mark for polycomb-mediated transcriptional repression was demonstrated using fly genetics. Pengelly and colleagues elegantly showed that *Drosophila,* harboring a point mutation in histone H3, which prevents PRC2-mediated methylation in histone H3 lysine 27, fails to repress PRC2-dependent genes [123]. Moreover, the mutant histone H3 cells recapitulated the homeotic transformations observed in mutant fly cells harboring a catalytic-inactive E(z) mutant (EZH2 orthologue), highlighting the importance of H3K27me3 for polycomb-mediated transcriptional repression [123]. At the molecular level, H3K27me3 deposition antagonizes the activating histone acetylation modification on histone H3 lysine 27 (H3K27ac) and interferes with the recruitment and activity of RNA polymerase II [124,125] (Figure 3A). Furthermore, H3K27me3 can facilitate the recruitment of cPRC1 to mediate repressive long-range chromatin interactions [40,41,60,126,127]. However, while cooperation between PRC1 and PRC2 ensures transcriptional repression of multiple polycomb target genes, PRC2-independent repression by PRC1 has been reported in several systems [39,76,109,128,129]. Finally, H3K27me3 deposited on parental nucleosomes is recognized by EED reader activity and enables the spreading of the mark by PRC2 to adjacent nucleosomes to facilitate the propagation of polycomb domains over large genomic distances [79,130]. The propagation of H3K27me3 during DNA replication from parental nucleosomes to daughter chromatin during DNA replication also enables the restoration of the epigenetic signature onto newly blank histones, thus maintaining a cellular memory of polycomb-mediated transcriptional repression throughout mitotic divisions [131,132,133,134]. 

### 3.3. Non-Catalytic Activities and Transcriptional Regulation by Polycomb Complexes

In addition to linear transcriptional regulation by polycomb complexes and their associated histone modifications, polycomb complexes play important roles in the regulation of 3D genomic architecture [104]. The colocalization of PRC1 and PRC2 typically results in compacted chromatin that can be observed as nuclear foci termed polycomb bodies, in which a higher-order chromatin organization of looping interactions and long-range interactions between individual polycomb domains are established [25,135,136] (Figure 3). This dynamic nuclear sub-clustering ranges from small neighboring genomic regions of 20–140 kilo-bases to distant genomic sites separated by mega-bases that represent the physical signature of polycomb-mediated transcriptional repression [25,126,137,138]. polycomb-mediated spatial regulation of chromatin architecture is governed by PRC1 complex activity and largely relies on the polymerization activity of the cPRC1 Polyhomeotic homolog (PHC) proteins via their sterile alpha motif (SAM) domain capable of establishing head-to-tail interactions that mediate the sub-nuclear clustering of PRC1 [127,139,140,141]. Mutations specifically within the SAM domain of PHC2 have clearly demonstrated the importance of cPRC1 polymerization activity in genomic clustering and the maintenance of stable genomic binding of PRC1 and PRC2 complexes [127]. Notably, although the recruitment of cPRC1 complexes is dependent to some extent on H2AK119ub-mediated recruitment and activity of PRC2 complexes, the higher-order organization, and chromatin compaction functions of cPRC1 were shown to be independent of PRC1 catalysis of H2AK119ub, directly linking cPRC1 non-catalytic activities to transcriptional repression [126,127,137,142,143]. 

## 4. The Role of PRC1 Complexes at Active Genes

While polycomb complexes are well-known for their prominent roles in transcriptional repression, accumulating evidence indicates that some PRC1-type PcG components are bound to active genes in flies and various mammalian cell types, including cancer cells [47,48,49,144,145,146,147,148]. Rather than repressing the expression of those actively transcribed genes, PRC1 seems to promote their expression, as impairment of PRC1 activity and binding results in the downregulation of those PRC1 targets [47,49,128,148]. In most of those cases, PRC1-bound active loci lack CBX proteins, PRC2 subunit binding, or H3K27me3, suggesting the presence of PRC2-independent ncPRC1 complexes at active genes. Indeed, genome-wide profiling of PRC1 components identified several key ncPRC1 subunits at active genes. In K562 leukemic cells, in addition to RING1B, ncPRC1.1 subunits, such as PCGF1 and KDM2B, are co-localized at active genes [145]. Studies in neuronal cells demonstrated that ncPRC1.5 key subunits such as AUTS2 and PCGF5 are localized to active genes [148], while in breast cancer cells RING1B co-localizes with ncPRC1.2 core subunit PCGF2 [144]. Finally, genome-wide profiling of PRC1 core subunits in epidermal SCs identified both overlapping and non-overlapping binding of several ncPRC1 complexes including ncPRC1.1, ncPRC1.4, and ncPRC1.6 at active genes [47]. However, paradoxically, while ncPRC1 complexes are highly proficient in catalysis of H2KA119ub, the epigenetic landscape at PRC1-bound active loci shows low to no H2AK119ub deposition, suggesting that ncPRC1 complexes promote gene expression through molecular mechanisms independently of their catalytic activity [47,48,144,145]. In line with this, recent studies have shown that the PRC1 catalysis of H2AK119ub is central for polycomb-mediated transcriptional repression, both in vitro [109,115] and in vivo [47]. Moreover, ectopic expression of either wild-type or catalytic-inactive RING1B in epidermal progenitors led to a similar increase in RING1B binding and a mild upregulation in the expression of active RING1B target genes [47]. 

How ncPRC1 complexes promote expression of their active target genes is not well understood, but it seems to involve both post-transcriptional modifications of ncPRC1 complex components to inhibit PRC1 catalytic activity as well as the cooperation with lineage-specific transcription factors. In quiescent lymphocytes, the Aurora B kinase (AURKB) cooperates with RING1B and inhibits its catalytic activity and H2AK119ub deposition by phosphorylating its E2 ubiquitin-conjugating enzyme, UBE2D3, and by phosphorylation-dependent activation of the histone de-ubiquitinase USP16 [49]. The nature of the cooperation between RING1B and AURKB acts to promote gene expression, and knockout of either one of these two proteins results in reduced RNA polymerase II binding and transcriptional down-regulation [49]. Similarly, in the mouse central nervous system, the phosphorylation of RING1B at serine 168 by casein kinase 2 inhibits the E3 ubiquitin ligase activity of RING1B, which, through cooperation with the neuronal transcription factor AUTS2, recruits the EP300 transcriptional co-activator to promote gene activation [148]. Examples for additional transcription factors that function together with ncPRC1 to promote gene activation in other cell types include TEX10, SALL4, ERα, GATA1, and bHLHE40 [106,144,149]. Overall, these fascinating and unexpected functions of ncPRC1 complexes at active genes are a major subject for further investigation and may explain to some extent the phenotypic discrepancies raised due to the ablation of PRC1 versus PRC2 global activities. 

## 5. Role of Polycomb Complexes in Adult Stem Cells

Epigenetic regulators, such as the PcGs of proteins, have been implicated in mediating versatile mechanisms that dictate changes in gene expression in adult SCs to maintain homeostatic and regenerative balances for tissue longevity. While the biological function of PRC1 and PRC2 has been extensively investigated during embryonic development, and in human and mouse embryonic SCs (ESCs), their functional role in adult SCs and in maintaining tissue homeostasis in vivo has become the focus of several studies in the past few years. Uncovering the functional significance of polycombs and dissecting the transcriptional axis controlled by polycombs in adult tissues will give us insight into how dysregulation of polycombs can lead to diseases such as cancer. In this section we will highlight some of those recent discoveries in several adult SCs and tissues.

### 5.1. Polycomb Complexes Are Required for Adult Intestinal Regeneration

The small intestine undergoes renewal every four to five days, making it one of the most dynamic adult tissues of the body. This high turnover is accomplished by fast-cycling intestinal stem cells (ISCs) that reside at the bottom of the intestinal crypts and express the R-spondin receptor LGR5 [150,151]. The LGR5 positive (+) ISCs divide symmetrically, to give rise to transit-amplifying (TA) progenitors that migrate towards the tip of the villi to differentiate into absorptive or secretory cells [152,153]. By using a mouse model that couples a constitutive null allele of *Ring1a* and a Cre-dependent conditional knockout (cKO) allele for *Ring1b* (Table 1), Chiacchiera and colleagues uncovered that inducible ablation of PRC1 function in the adult intestine results in a rapid reduction of body weight, thinner intestine, and degenerating crypt architecture [22]. Notably, loss of PRC1 and H2AK119ub does not affect the global deposition of H3K27me3, indicating that PRC1 function is independent of PRC2 in the adult intestinal crypts. They also observed fewer number of LGR5+ ISCs that failed to complete differentiation, suggesting that PRC1 plays a role in controlling intestinal homeostasis and regeneration. Particularly, the loss of crypt architecture was not a result of apoptosis but was associated with reduced ISC proliferation and self-renewal. To gain insight into this phenotype, the authors performed RNA-seq analysis, which revealed that most of the differentially expressed genes in PRC1-null ISCs, compared with control ISCs, were upregulated and did not belong to the intestinal lineage. Many of those upregulated genes were direct PRC1 targets demonstrating the importance of transcriptional repression by PRC1 in the intestine. A gene ontology analysis revealed that DNA-binding transcription factors (TFs), notably the ZIC family of TFs, were significantly enriched among the upregulated genes. The ZIC TFs are negative regulators of the Wnt signaling pathway, known to suppress the Wnt/β-catenin transcriptional axis [154,155]. The Wnt signaling pathway has been shown to be essential for ISC self-renewal and regeneration of the intestine [156]. By using a combination of ectopic expression/inactivation of ZICs, and immunoprecipitation experiments in cancer cell lines and intestinal crypt-derived organoids, the authors showed that ZIC1 and ZIC2 inhibits Wnt signaling by physically interacting with the β-catenin/TCF complex. Altogether, this study showed that PRC1 maintains ISC self-renewal and overall intestinal homeostasis by repressing the expression of ZIC TFs to sustain Wnt signaling in ISCs. 

In addition to the fast-cycling LGR5+ ISCs, there are also slow cycling BMI1 expressing ISCs present in the small intestine that have been shown to give rise to LGR5+ ISCs following an injury [168]. Dun et al., reported that while BMI1 is abundantly expressed in the small intestine of young and adult rats, its expression is significantly reduced in aging rats coinciding with decreased proliferation of crypt cells in aging rats [169]. Given that the Wnt/β-catenin pathway is imperative for ISC proliferation, and BMI1 is known to promote the Wnt signaling pathway by repressing Dkk1, a Wnt pathway inhibitor, the authors sought to define the expression of β-catenin in the small intestine of ageing rats [170,171,172]. In line with reduced BMI1 expression, the authors observed downregulation of canonical Wnt signaling pathway members in addition to the increased expression of Dkk1 in the small intestine of ageing animals. These observations suggest that PRC1 activity may play a role in regenerative capacities within the aging intestine, however further functional studies are warranted to test this possibility. Although, these observations are correlative, it suggests that regulation of PRC1 and pathways directly under its control has implications in slower regenerative capacities associated with aging. 

In contrast to the essential role of PRC1 in ISCs, Chiacchiera et al., reported that conditional ablation of PRC2 core subunit *Eed*, in the small intestine, by utilizing the *AhCre*;*Eed^fl/fl^* mouse model (Table 1), is dispensable for intestinal regeneration [157]. Loss of *Eed* leads to impaired H3K27me3 deposition but does not alter PRC1-dependent H2AK119ub in intestinal epithelial cells, suggesting that loss of PRC2 does not affect PRC1 catalytic activity in the ISCs. Lineage tracing of *Eed*-null ISCs showed that fast-cycling renewal of ISCs remain unperturbed upon *Eed* loss, but crypt architecture was altered coupled with defects in cell proliferation of TACs. Notably, loss of *Eed* leads to an increase in goblet cells and enteroendocrine cells, suggesting that the ablation of PRC2 activity affects the secretory lineage commitment without altering global enterocytic differentiation. However, all these changes cumulatively did not affect the homeostatic regeneration of the organ. RNA-seq analysis of the TACs isolated from the crypts revealed upregulation of genes involved in cell cycle arrest and expansion of goblet cells which belong to the secretory lineage. Moreover, RNA-seq and ChIP-seq analyses of wildtype and *Eed*-null crypt cells show that TFs, such as *Atoh1* and *Spdef*, which are known to promote goblet cell differentiation, are significantly upregulated in the TACs and are demarcated with H3K27me3. Contrary to studies reported by Chiacchiera et al, Koppens and colleagues showed that loss of PRC2 in ISCs leads to significantly lower body weight, when compared with control mice, and to defects in overall homeostatic regulation of the intestinal epithelium [20,157]. Using similar conditional ablation of *Eed* strategy in ISCs (Table 1) they showed that *Eed* cKO crypts had severely defective crypt architecture, the affected crypts underwent necrosis and microcystic degeneration, and a small number of crypts showed hypertrophy and hyperplasia. Similar to the previous study, Koppens et al. showed that *Eed c*KO crypt cells had upregulated expression of *Cdnk2a* and reduced proliferation. They also reported a defect in the differentiation program in *Eed c*KO intestines, where the differentiation of secretory cell types was increased compared with any other lineages. However, the major conflicting observation between the two studies is that, while Chiacchiera et al. reported that PRC2 function is dispensable for ISC self-renewal, Koppens et al. reported that loss of *Eed* leads to dramatic loss of Lgr5+ ISCs. The discrepancy between two studies was discussed in Jadhav et al. [173]. The authors reported that ablation of PRC2 in ISCs does not lead to immediate loss of H3K27me3 and about 40% of residual H3K27me3 levels were maintained, keeping target genes repressed. Using ChIP and computational strategies this group showed that H3K27me3 dilutes parental nucleosomes only, upon cell division, and it takes several rounds of division to achieve the full loss of H3K27me3 and the consequent activation of genes under PRC2 repression. Therefore, this observation might explain the opposing consequences of loss of PRC2 function in the mouse intestines and the tissue defects arise from varying number of PRC2-null ISC divisions. In line with the similarities between phenotypic alterations upon intestine deletion of either PRC1 or PRC2 complex activity, molecular studies by Koppens et al. have demonstrated that both complexes co-regulate the Wnt signaling pathway in the intestine [20,22]. Using RNA-seq and ChIP-seq experiments, they showed that several Wnt target genes, including *Ascl2*, *Axin2* and *Lgr5,* were downregulated upon loss of PRC2. Notably, ZIC TFs, which have also been shown to be upregulated in PRC1-null intestines [22], were also upregulated in the *Eed*-null crypts, suggesting that the Wnt signaling pathway is abrogated upon the loss of PRC2 function, leading to the severe defects discussed. ChIP-seq experiments with H3K27me3 have revealed that almost half of the genes upregulated after *Eed* ablation are marked by H3K27me3 and thus are direct targets of PRC2. Additionally, a comparison with H2AK119ub ChIP-seq, done in an earlier study, revealed that the majority of the H3K27me3-associated genes were enriched with H2AK119ub, indicating that the genes required for ISC maintenance are under concerted control of both PRC1 and PRC2. Altogether, these studies highlight that both PRC1 and PRC2 work in concert to maintain adult intestinal homeostasis and regeneration. 

### 5.2. Polycomb Complexes Play a Critical Role in Adult Hematopoiesis 

Hematopoietic stem cells (HSCs) give rise to a diverse array of cells of the blood lineage [174]. The polycomb machinery has been shown to maintain self-renewal capacity of HSCs by repressing expression of tumor suppressor genes, maintain redox homeostasis in HSCs to prevent their premature loss, and promote lineage commitment in HSC progenitors for proper bone marrow cellularity [18,21,23,175,176,177,178]. The role of polycomb complexes in adult HSC maintenance, self-renewalm and differentiation has been thoroughly studied over the past two decades and was recently summarized in an extensive review [18,179]. In this section we will discuss the recent discoveries that were not covered by Di Carlo et al.

The PRC1 subunit, BMI1, was shown to play an essential role in maintaining self-renewal of HSCs by repressing the *Ink4a/Arf* locus, which encodes for the cell cycle inhibitor p16^Ink4a^ and the tumor suppressor p19^Arf^ [180,181]. Additionally, constitutive expression of BMI1 enhances self-renewal capacity of HSCs and confers stress resistance to HSCs during serial transplantation [182]. In line with these observations, Nitta et al. showed that overexpressing BMI1 in hematopoietic cells led to an expansion of myeloid-committed progenitors and age-related anemia was significantly reduced in adult mice [159]. Transplantation of BMI1 overexpressing HSCs and progenitors from aged mice were successfully able to undergo hematopoietic reconstitution much better than cells from age-matched controls, indicating that sustained BMI1 expression is able to counteract aging stressors that often lead to lower repopulating capacities of HSCs. RNA-sequencing analysis of control and sustained BMI1-expressing HSCs and progenitor cells, from young and aged mice, revealed that the sustained expression of BMI1 represses genes that get de-repressed upon aging. Moreover, sustained expression of BMI1 also upregulated the expression of HSC genes that are downregulated in aged HSCs. Together, these observations indicate that BMI1 expression in HSCs protects the cells against gradual loss of stemness that occurs during aging. Collectively, this work highlights that manipulation of BMI1 expression could be a potential therapeutic approach to combat the age-related decline of HSC function.

Bone marrow stromal cells (BMSCs) are multipotent progenitor cells that form osteoblasts and adipocytes and are components of the perivascular HSC niche [183]. Hu et al. investigated the role of BMI1 in regulation of BMSCs [158]. The authors showed that BMI1 is expressed at high levels in BMSCs, and its levels decreases in BMSCs cells undergoing adipocyte differentiation. Passaging of wild-type and *Bmi1*-null BMSCs revealed that proliferative capacity of *Bmi1*-null BMSCs is severely reduced and exhibited increased senescence with elevated *Ink4a* expression in vitro. Additionally, differentiation stimulation revealed increased adipocyte differentiation and increased expression of genes associated with adipogenesis in BMSCs from *Bmi1*-null mice when compared with controls. Additionally, *Bmi1*-null mice exhibited reduction in bone marrow cellularity and hematopoietic progenitor cells. Overall, the findings in this study revealed that BMI1 function is required to suppress adipogenic differentiation in BMSCs to maintain adult bone marrow HSCs. 

Ikeda et al., investigated the role of *Eed* in adult hematopoiesis by generated a mouse model by crossing *Eed^flox/flox^* with *Cre^ERT2+^* to conditionally ablate *Eed* function in adult mice upon tamoxifen administration (Table 1) [160]. Loss of *Eed* in adult mice resulted in premature death coupled with reduction in all hematopoietic lineages, hemoglobin concentration, and platelet numbers. *Eed*-null mice also presented thymic and splenic atrophy and pale appearance of bone marrow indicating that *Eed* plays an essential role in maintaining adult hematopoiesis. Moreover, bone marrow transplantation assays revealed that HSCs and its progenitors from *Eed*-null mice failed to reconstitute and resulted in a reduction in HSCs, progenitors, and myeloid fractions in the donor mice. RNA-seq studies showed that *Eed*-null HSCs significantly upregulated cell adhesion molecules, including integrins, cadherins, selectins, and claudins. Given that fibronectin, which is a major component of the hematopoietic niche, interacts with integrins [184], the authors tested if *Eed*-null HSCs had higher adhesion to fibronectin. Indeed, *Eed*-null HSCs exhibited significantly higher adhesion to fibronectin compared with controls, thus suggesting that *Eed* loss promotes the HSCs to remain bound to the HSC niche. Lastly, the authors also report the upregulation of several pathways in *Eed*-null HSCs that are involved in leukemogenesis, indicating that dysregulation of *Eed* might contribute to myelodysplastic and leukemic predisposition. Given that PRC2 is dysregulated in several hematological malignancies [185], these functional in vivo studies provide further evidence on the importance of PRC2 in maintaining proper hematopoietic function in adults. 

PHD finger protein 19 (Phf19) is PRC2-associated factor that has been shown to modulate the enzymatic activity of PRC2 in mouse ESCs (mESC) [87,88]. Phf19 expression declines during mESC differentiation and has been shown to have high expression in adult stem cell compartments, including ISCs [186,187]. However, its functional role in adult stem cell systems remains to be investigated thoroughly. Vizán et al., reported that in hematopoietic system, Phf19 expression is elevated in undifferentiated progenitors and progressively decreases upon differentiation [161]. To investigate the role of *Phf19* in HSCs, the authors generated a *Phf19* KO mouse model (Table 1), where Phf19 function was abrogated in all tissues. Even though the young adult KO mice did not exhibit any major organ dysfunction, they did present anterior-to-posterior homeotic transformation, in line with observations made in other polycomb member-deficient mice [188]. Aged *Phf19* KO mice exhibited high penetrance of splenomegaly, a reduction in HSCs and decrease in the number of actively dividing HSCs. Bone marrow transplantation experiments showed that *Phf19* KO HSCs failed to reconstitute cellular lineages, indicating that the loss of Phf19 leads to HSC malfunction. Transcriptome analysis revealed that the Myc transcriptional network, which is required for HSC differentiation was downregulated in Phf19 KO HSCs, along with the downregulation of biosynthetic pathways supporting the observation that loss of Phf19 in HSCs leads to enhanced quiescence, a hallmark of aged HSCs. Interestingly, comparative analysis between Phf19 KO HSCs and aged HSCs revealed that the KO cells strongly resembles aged HSCs that seem to accumulate but fail to generate new blood cells in aged animals. Additionally, ChIP-seq studies revealed that H3K27me3 is increased in Phf19-null HSCs, which is also a hallmark of aged HSCs. Altogether, the data in this study highlight the function of a PRC2-associated protein in adult hematopoiesis and how its dysregulation has implications in enforced HSC quiescence, which is often seen in aged HSCs and relapsed leukemia.

### 5.3. Role of Polycomb Complexes in Adult Epidermal Stem Cells 

The skin is the largest organ of the body and serves as the first protective barrier, therefore requiring a stringent homeostatic and regenerative program. The mammalian epidermis is composed of the interfollicular epidermis and the pilosebaceous unit, which includes the hair follicle (HF) and the sebaceous gland [189]. During development a consortium of SCs specify these epidermal compartments and their regulation is orchestrated several chromatin regulators [190,191]. Especially, the role of polycomb complexes and the role of specific polycomb proteins have been extensively studied in the developing skin [39,47,108,128,192,193]. However, their role in the resident SCs in all the adult epidermal compartments that aide in maintaining overall homeostasis and regeneration of the tissue is understudied. Hair regeneration in the adult skin occurs in a cyclical manner and is mediated by two different pools of SCs located in the bottom most part of the HF; the CD34+ hair follicle stem cells (HFSCs), located in the bulge, and the LGR5+ HFSCs, located in the hair germ (HG) [189,194,195,196]. While CD34+ HFSCs are long-lived SCs that fuel hair growth throughout the life of an organism, LGR5+ cells are short-lived committed progenitors that generate differentiated HF layers, making them imperative for the process of hair regeneration [195,197,198,199]. Pivetti et al., showed that loss of PRC1 function in the LGR5+ cells in adult mice results in severely delayed hair regeneration, with hair follicles arrested at the earliest stages of hair growth (Table 1) [164]. Lineage tracing experiments further revealed that, in line with the hair growth-arrest phenotype, PRC1-null LGR5+ cell numbers were markedly reduced. Transcriptional analysis of control and PRC1-null LGR5+ cells revealed that pathways associated with general developmental processes were upregulated and several non-lineage specific genes were transcriptionally activated. This observation was in line with what the group had observed with ISCs (discussed above). A comparison of the transcriptional profiles of PRC1-null ISCs and LGR5+ cells revealed that 25% of upregulated genes were common in both SCs and comprised of mainly homeobox-containing TFs involved in development. Additionally, ChIP-seq for RING1B in LGR5+ cells revealed that RING1B distribution was similar to that observed in ISCs, highlighting that PRC1 has a conserved role of maintaining lineage identity in SCs of different adult tissues [22]. However, it is important to note that not all the PRC1-bound targets become activated in PRC1-null LGR5+ cells and ISCs and the resulting transcriptional landscape upon the loss of PRC1 is context-dependent. 

The loss of PRC2 in the developing epidermis has various phenotypic consequences on epidermal barrier formation and HF morphogenesis [39,108,192], but, until recently, we had limited knowledge of its physiological function in the adult skin. Majetta and their group have reported that the loss of function of JARID2, a key PRC2.2 complex member, in the adult epidermis leads to decreased basal cell proliferation, but enhanced differentiation indicated by thick suprabasal layers (Table 1). Additionally, JARID2-null mice display a significant delay in the proliferation of hair germ cells during the onset of hair growth but does not abrogate hair cycle progression. Notably, loss of JARID2 leads to lower H3K27me3 and increased expression of *Ink4a* in the adult interfollicular epidermis but does not translate into gross phenotypic changes [163], suggesting that the PRC2.2 complex does not have an instructive role in the adult epidermis. Recently, Li et al., have reported that low-dose ultraviolet B (UVB) exposure leads to the downregulation of PcG of proteins, EED and RING1B, and their associated repressive marks H3K27me3 and H2AK119ub in the interfollicular epidermis (IFE) [162]. Interestingly, individual conditional ablation of PRC1 and PRC2 function leads to the same phenotype of epidermal pigmentation, indicating that the polycomb complexes work in a canonical fashion in epidermal stem cells (EpSCs). Transmission electron microscopy revealed the presence of melanosomes, in both keratinocytes and melanocytes of PRC2-null skin, a phenomenon that was not observed in control mice. Moreover, ablation of both PRC1 and PRC2 function leads to the migration and trans-location of melanocytes from its homeostatic niche located at HF bulge to the IFE. ChIP-seq analysis of H3K27me3 and H2AK119ub combined with RNA-seq analysis of PRC1-, PRC2-null, and control EpSCs revealed that both complexes co-repress genes that are known to be upregulated upon UVB irradiation. Notably, these genes have been linked to control melanocyte behavior, reealing the phenomenon that polycomb represses UVB-induced genes in the EpSCs that control melanocyte behavior and function. Further analysis identified that genes upregulated in both PRC1-null and PRC2-null EpSCs are associated with protein secretion and extracellular matrix (ECM) organization, including collagen II (COL2A1) that this study has identified to be an important regulator of melanogenesis. Taken together Li et al., identified that the polycomb-*Col2a1* transcriptional axis is a major regulator of epidermal pigmentation upon UV exposure. 

Several correlative studies in the recent years suggest that PRC2 may be playing a role in the adult CD34+ HFSCs. Lien et al., conducted genome-wide mapping of H3K27me3 in quiescent and activated CD34+ HFSCs, as well as in HF-TACs, and showed that H3K27me3 demarcates key HF-TAC genes in the HFSCs and this mark is lost from these genes in the HF-TACs [200]. These findings imply that PRC2-mediated gene repression via H3K27me3 might be involved in maintaining HFSC cell identity. Additionally, Lee et al., showed that during homeostatic hair cycle levels of H3K27me3, H3K4me3 and H3K9me3 marks are reduced in quiescent HFSCs compared to proliferative HFSCs, indicating that this dynamic chromatin distribution is coupled to increased HFSC plasticity prior to activation [201]. Pharmacological inhibition of de-methylases in the skin, including the HFSCs, resulted in elevated levels of global tri-methylation and defective hair regeneration. Follow-up studies, by Kang et al., further expanded on this finding and showed that failure to modulate global tri-methylation at proper hair-cycle stages results in defective re-epithelization following injury [202]. Future studies showing functional relevance of PRC2 and H3K27me3 in the HFSCs will uncover its direct role in hair regeneration. 

### 5.4. Polycomb Group Proteins Are Expressed in the Adult Olfactory Epithelium 

The olfactory epithelium (OE) has long served as a model in which to study adult neurogenesis and neuroepithelial renewal [203]. The OE consists of the olfactory receptor neurons, sustentacular and microvillar supporting cells, and basal cells [204,205,206]. The SC and progenitor cells of the OE reside in the basal germinal zone [207,208]. The OE basal cells comprise of the quiescent horizontal basal cells (HBCs) and the heterogenous subsets of proliferative globose basal cells (GBCs) [209]. These cells regenerate and replace mature cell types throughout the lifetime of an organism and there has been much interest in understanding how the OE basal cell maintenance and homeostasis are regulated. Goldstein et al., showed that while BMI1 was expressed in GBCs and mature neurons, the expression of PRC2 complex proteins EZH2 and SUZ12 remained confined to the GBCs of the OE [210]. Interestingly, following chemical lesion which removes most of the OE epithelium, the authors found BMI1+ cells in the remaining thin layer of the OE, and within 7 days post-lesion, BMI1+ progenitors were present in the regenerating OE, indicating that BMI1+ cells contribute to epithelial reconstitution. Further analysis of un-lesioned wild-type OE tissue revealed that BMI1+ GBCs were located above the HBC layer and were co-labeled with SOX2, a maker of multipotent GBCs that give rise to TACs [205]. Knockdown of *Bmi1* in cultures did lead to changes in gene expression but did not result in rapid phenotypic changes. However, pharmacological inhibition of EZH2 in cultures resulted in proliferation defects. While the previous study by Goldstein et al. focused on BMI1 expression and function in the OE, their followup studies were focused on the expression and function of other PcG proteins in the regenerating OE. They show that post chemical lesion, PRC2 complex member EZH2 is expressed in c-KIT+ cells which are indicative of proliferative SCs and progenitor cells [211]. This reinstated that PRC2-expressing basal SCs of the OE are active and contribute to OE regeneration following injury, similar to that of BMI1+ cells. They also reported that, while BMI1 expression was more widespread in their compartments of the OE, the expression of MEL18, another PRC1 component, was confined to the nuclei of GBCs and immature neurons. Additionally, expression of CBX8, a canonical PRC1 member, was confined to the neuronal lineage cells of the OE, suggesting that it may be regulating PRC1 targeting in the neuronal-fated cells to confer lineage specificity [212]. Altogether, these studies suggest that polycomb-mediated epigenetic regulation in the OE basal SCs and its progenitor might have a crucial role in the adult OE and further loss-of-function studies need to be conducted in vivo to ascertain if the PcGs of proteins mediate OE basal SC self-renewal and organ regeneration. 

### 5.5. The Role of Polycomb Repressive Complex 1 in Other Adult Stem Cell Systems

The mammalian adult heart was thought to be a terminally differentiated organ with no regenerative capacity, but studies from the last decade have identified a reservoir of cardiac stem cells (CSCs) that maintains a low cardiomyocyte (CM) turnover during heart homeostasis [213,214,215]. Valiente-Alandi and colleagues reported that a subpopulation of Sca1+ cardiac progenitor cells (CPCs) express BMI1 (BMI1-CPCs) [216]. Lineage-tracing experiments, coupled with fluorescence-activated cell sorting (FACS), confirmed that these BMI1-CPCs were not only maintained but expanded in aged mice, indicating their contribution towards several cardiac lineages. Lastly, the authors establish that these cells are non-CMs and distinct cardiac populations that express high levels of stemness and cardiac lineage specification markers capable of self-maintenance. The follow-up study by this group showed that BMI1-derived cells give rise to CMs after myocardial infarction, establishing the observation that BMI1 expressing CPCs are the source of progenitors that aide in cardiac repair [217]. Moreover, RNA-seq analysis of BMI1-CPCs, five days after injury, revealed that these cells upregulate genes related to cell proliferation, cycles, and migration to promote repair. Lastly, work done by Herrero and colleagues showed that loss of BMI1-CPCs does not affect cardiac function in steady state, following an acute myocardial infarction. However, BMI1-CPC-deficient hearts had clear cardiac remodeling defects along with prolonged cardiac dysfunction and deficient angiogenesis when compared with infarcted controls [218]. Together these studies have not only established that the adult mammalian heart have a distinct BMI1-expressing progenitor cell population, but also have identified that these cells are required for proper angiogenic response following an injury. Further work needs to be done to identify the functional role of *Bmi1* in these specialized cells to promote its tissue-replenishing capacity. 

Mesenchymal stem cells (MSCs) are multipotent cells that have high self-renewal and proliferative potential, capable of differentiating into multiple cell lineages [219,220]. The continuously growing adult mouse incisor serves as an excellent model to investigate the organization of adult MSC niches. In the tooth, the MSCs reside in the dental pulp and give rise to transit-amplifying cells (TACs) that rapidly proliferate and differentiate into specialized tooth-specific cell types [219,221,222]. In adults, growth homeostasis is achieved by the rate off transition of MSCs to TACs and the subsequent rate of proliferation and differentiation of the TACs [219,221,222]. Notably, BMI1 is expressed in the incisor SCs and the loss of BMI1 leads to reduced stem cell numbers in the incisor and defective enamel production. Transcriptional studies in *Bmi1*-null mice revealed that BMI1 represses the *Hox* genes in the adult incisor, which is upregulated in *Bmi1*-null mice, leading to the transcription of genes associated with premature differentiation (Table 1) [167]. Additionally, RING1B expression is localized in mesenchymal cells, with the highest rates of proliferation in the mouse incisor, and the targeted deletion of PRC1 function in these cells leads to the loss of TAC proliferation and arrested incisor growth [165]. An et al. expanded on this observation and showed that RING1B protein is specifically localized in the rapidly proliferating MSCs of the incisor, which are located distally to the slow-cycling MSCs [166]. Transcriptional microarray analysis of wildtype and PRC1-null mesenchymal pulp cells revealed that loss of PRC1 function leads to the upregulation of Hox and cell-cycle inhibitor genes and the downregulation of pathways associated to cell proliferation, keeping in line with the observation that loss of PRC1 leads to decreased cell proliferation of TACs (Table 1). Furthermore, ChIP-seq analysis identified *Cdkn2a*, a major negative regulator of cell cycles, to be a direct target of RING1B and H3K27me3. The microarray analysis also revealed downregulation of the Wnt pathway in PRC1-null TACs. Interestingly, ChIP-seq analysis had revealed that ZIC1 and ZIC2, was bound by RING1B. Therefore, highlighting that PRC1 regulates the Wnt pathway, which is involved in cell proliferation, stem-cell renewal, and cell specification in several tissues, by suppressing the ZIC1 and ZIC2 TFs [154,155]. The authors also report that loss of PRC1 in TACs leads to apoptosis of the slow-cycling MSCs, suggesting that the TACs provide a supportive environment to the MSCs by secreting factors that act as positive regulator of the MSCs. Given that the loss of Wnt signaling also leads to the apoptosis of SCs [223], cumulatively the observations made in this study indicate that Wnt activity downstream of PRC1 in TACs is required for MSC maintenance. 

## 6. Conclusions and Future Perspectives

Understanding the mechanisms controlling tissue- and stage-specific transcriptional programs remains a major challenge in biology. Over the last two decades, the PcGs of proteins have emerged as key regulators of adult stem cells and tissue homeostasis [179,224,225,226]. Canonical polycomb components, such as EZH2 or BMI1, have been reported to be enriched in the stem cell compartment of several tissues including the intestine, skin, tongue, cardiac progenitors, and hematopoietic stem cells [23,116,117,168,216,227]. In some of these adult tissues, the proliferative and regenerative capacity of the stem cell compartment is associated with the expression of key PcG subunits, while, in other tissues, functional studies have clearly established a role for polycomb complexes in these processes. In addition, polycomb complexes play an important role in preserving stem cell transcriptional identity and lineage fidelity throughout mitotic division by the repression of unwanted genes [27,134,228,229]. Two nice examples of this are the restriction of the Merkel cell lineage in the developing skin epithelium [47,108], and the conversion of T cells into B cells in the absence of PRC1 activity, or B1 to B2 cells in the absence of RYBP activity, in hematopoietic cells [160,230].

Recent advances in the field indicate that the molecular mechanisms by which polycomb complexes regulate spatiotemporal gene expression patterns are much more intricate than originally envisioned. Biochemical studies have uncovered that there are various PRC1-type and PRC2-type complexes that, through interaction with multiple accessory subunits, acquire cell- and context-specific functions [44,48,77,84]. Thus, future study dissecting the roles of specific polycomb complexes in adult tissues is much needed, in order to grasp the full spectrum of polycombs’ activities in adult stem cells. Since multiple studies report alterations in PcG subunits in various types of cancer [231,232,233], identifying the complex-specific polycomb functions and molecular pathways is not only important for the understanding of how adult tissue homeostasis is epigenetically regulated, but also for the understanding of tissue tumorigenesis. 

## Figures and Tables

**Table 1:** Role of PcGs of proteins in adult SC compartments.

Tissue Studied	Targeted Gene	Mouse Model Used	Observed Phenotypes	Biological Process Affected	References
Intestine	*Ring1a/b*	*Ring1a^−/−^*;*Ring1b^fl/fl^*; *LGR5-eGFP-IRES-Cre^ERT2^*	lower body weight, thinner intestine, defect in crypt architecture	reduced self-renewal and proliferation of intestinal stem cells, expression of non-lineage transcription factors	Chiacchiera et al., 2016 [22]
	*Eed*	*Eed^fl/fl^*; *AhCre*	alteration in crypt-villus architecture, expansion of goblet cell and enteroendocrine cells	reduced proliferation of transit amplifying cells, lowered secretory lineage commitment	Chiacchiera et al., 2016 [157]
	*Eed*	*Eed^fl/fl^*; *AhCre*	lower body weight, extensive crypt and microcystic degeneration, necrosis, hypertrophy, and hyperplasia of crypts	reduced proliferation of intestinal crypt cells	Koppens et al., 2016 [20]
Bone marrow stromal cells	*Bmi1*	*Bmi1^fl/fl^*; *Prx1-Cre*	accumulation of bone marrow adipocytes, reduction in bone marrow cellularity	increased senescence, depletion of HSCs and progenitor cells, increased adipogenic differentiation	Hu et al., 2019 [158]
Hematopoietic cells	*Bmi1*	*Vav-Cre*;*Rosa26Stop^FL^Bmi1* *(over-expression of Bmi1)*	reduction in age-related anemia	attenuated age-related HSC function, maintenance of HSC signature gene expression	Nitta et al., 2020 [159]
	*Eed*	*Eed^fl/fl^*; *Cre^ERT2^*	thymic and splenic atrophy, pale bone marrow, hematopoietic dysplasia	reduction in all hematopoietic cellular lineages, abnormal cell cycle, upregulation of adhesion molecule genes	Ikeda et al., 2016 [160]
	*Phf19*	*Phf19*^−^*/*^−^**	high penetrance of splenomegaly	upregulation of retinoic acid pathway, downregulation of Myc network and genes related to biosynthesis and energy production	Vizán et al., 2020 [161]
Interfollicular epidermis	*Eed* and *Ring1a/b*	*Eed^fl/fl^*; *K14-Cre^ERT2^* and Ring1a^−/−^;Ring1b^fl/fl^; K14-Cre^ERT2^	epidermal pigmentation	upregulation of UV-responsive genes in EpSCs, induction of COL2A1 expression which promotes epidermal pigmentation	Li et al., 2021 [162]
Interfollicular epidermis and hair follicle	*Jarid2*	*Jarid2^fl/fl^*; *K14CreYFP::Rosa26*	defective epidermal thickness and delayed hair cycle	reduced proliferation of basal cells of the epidermis and hair germ cells of the hair follicle, enhanced differentiation of basal cells	Mejetta et al., 2011 [163]
Hair follicle	*Ring1a/b*	*Ring1a^−/−^*;*Ring1b^fl/fl^*; *LGR5-eGFP-IRES-Cre^ERT2^*	arrested hair follicle growth	defective Lgr5+ HFSC differentiation due to upregulation of non-lineage genes	Pivetti et al., 2019 [164]
Incisors	*Ring1a/b*	*Ring1a^−/−^*;*Ring1b^fl/fl^*; *Rosa26::Cre^ERT2^*	Defective enamel and dentin formation	Reduced proliferation of mesenchymal transit amplifying cells	Lapthanasupkul et al., 2012 [165], An et al., 2018 [166]
	*Bmi1*	*Bmi1^GFP/GFP^* (*Bmi1-*null mice)	defective enamel production	fewer stem cells due to upregulation of *Ink4a/Arf,* upregulation of *Hox* genes leading to premature differentiation and loss of stem cell population	Biehs et al., 2013 [167]

## Data Availability

Not applicable.
